# Adherence to antipsychotic laboratory monitoring guidelines in children and youth: a population-based study

**DOI:** 10.3389/fpsyt.2023.1172559

**Published:** 2023-05-12

**Authors:** Tony Antoniou, Tianru Wang, Kathleen Pajer, William Gardner, Yona Lunsky, Melanie Penner, Mina Tadrous, Muhammad Mamdani, David N. Juurlink, Tara Gomes

**Affiliations:** ^1^Li Ka Shing Knowledge Institute, St. Michael’s Hospital, Toronto, ON, Canada; ^2^Institute for Clinical Evaluative Sciences, Toronto, ON, Canada; ^3^Department of Family and Community Medicine, University of Toronto, Toronto, ON, Canada; ^4^Department of Family and Community Medicine, St. Michael’s Hospital, Toronto, ON, Canada; ^5^Children’s Hospital of Eastern Ontario Research Institute, Ottawa, ON, Canada; ^6^Department of Psychiatry, University of Ottawa, Ottawa, ON, Canada; ^7^School of Epidemiology and Public Health, University of Ottawa, Ottawa, ON, Canada; ^8^Azrieli Adult Neurodevelopmental Centre, Centre for Addiction and Mental Health, Toronto, ON, Canada; ^9^Department of Psychiatry, University of Toronto, Toronto, ON, Canada; ^10^Autism Research Centre, Bloorview Research Institute, Holland Bloorview Kids Rehabilitation Hospital, Toronto, ON, Canada; ^11^Department of Pediatrics, University of Toronto, Toronto ON, Canada; ^12^Leslie Dan Faculty of Pharmacy, University of Toronto, Toronto, ON, Canada; ^13^Li Ka Shing Centre for Healthcare Analytics Research and Training, Unity Health Toronto, Toronto, ON, Canada; ^14^Temerty Faculty of Medicine, University of Toronto, Toronto, ON, Canada; ^15^Institute of Health Policy, Management, and Evaluation, University of Toronto, Toronto, ON, Canada; ^16^Department of Medicine, University of Toronto, Toronto, ON, Canada

**Keywords:** antipsychotic agents/adverse effects, drug monitoring, adolescent, child, guidelines as topic

## Abstract

**Background:**

In 2011, the Canadian Alliance for Monitoring Effectiveness and Safety of Antipsychotics in Children (CAMESA) published guidelines for the metabolic monitoring of antipsychotic-treated children and youth. Population-based studies examining adherence to these guidelines are needed to ensure the safe use of antipsychotics in children and youth.

**Methods:**

We conducted a population-based study of all Ontario residents aged 0 to 24 who were newly dispensed an antipsychotic between April 1, 2018, and March 31, 2019. We estimated prevalence ratios (PRs) and 95% confidence intervals (CI) associating sociodemographic characteristics with the receipt of baseline and follow-up (3- and 6-month) laboratory testing using log-Poisson regression models.

**Results:**

Overall, 6,505 of 27,718 (23.5%) children and youth newly dispensed an antipsychotic received at least one guideline-recommended baseline test. Monitoring was more prevalent among individuals aged 10 to 14 years (PR 1.20; 95% CI 1.04 to 1.38), 15 to 19 years (PR 1.60; 95% CI 1.41 to 1.82), and 20 to 24 years (PR 1.71; 95% CI 1.50 to 1.94) compared to children under the age of 10. Baseline monitoring was associated with mental health-related hospitalizations or emergency department visits in the year preceding therapy (PR 1.76; 95% CI 1.65 to 1.87), a prior diagnosis of schizophrenia (PR 1.20; 95% CI 1.14 to 1.26) or diabetes (PR 1.35; 95% CI 1.19 to 1.54), benzodiazepine use (PR 1.13; 95% CI 1.04 to 1.24), and receipt of a prescription from a child and adolescent psychiatrist or developmental pediatrician versus a family physician (PR 1.41; 95% CI 1.34 to 1.48). Conversely, monitoring was less frequent in individuals co-prescribed stimulants (PR 0.83; 95% CI 0.75 to 0.91). The prevalence of any 3- and 6-month follow-up monitoring among children and youth receiving continuous antipsychotic therapy at these time points was 13.0% (1,179 of 9,080) and 11.4% (597 of 5,261), respectively. Correlates of follow-up testing were similar to those of baseline monitoring.

**Conclusion:**

Most children initiating antipsychotic therapy do not receive guideline-recommended metabolic laboratory monitoring. Further research is needed to understand reasons for poor guideline adherence and the role of clinician training and collaborative service models in promoting best monitoring practices.

## Introduction

Children and youth receiving treatment with antipsychotic drugs are at risk of various metabolic disorders, including hyperglycemia, dyslipidemia, and hyperprolactinemia (1, 2). In addition to their association with new-onset type 2 diabetes and sudden death, adverse metabolic effects and metabolic syndrome during childhood also predict increased adult cardiovascular risk (3–8). Therefore, early detection and management of adverse metabolic effects are required for children and youth treated with antipsychotics.

Several practice guidelines have been published for the metabolic monitoring of antipsychotic-treated children and youth (9–14). Generally, the guidelines are in agreement with the need for baseline and follow-up monitoring, with some variation in recommended follow-up intervals at which testing should be performed and the specific tests recommended. In 2011, the Canadian Alliance for Monitoring Effectiveness and Safety of Antipsychotics in Children (CAMESA) published recommendations for monitoring antipsychotic adverse effects in children and youth (15). Generally, these guidelines recommend monitoring of specified anthropomorphic and metabolic parameters, including laboratory assessments of glucose, lipids, serum transaminases, and prolactin at baseline and following 3- and 6-months of treatment, with some recommendations varying according to the specific antipsychotic drug used (15). However, despite the increased use of these medications over time, several studies have found low adherence to CAMESA’s laboratory monitoring recommendations. Specifically, a retrospective chart review of 294 antipsychotic-treated children and adolescents found that baseline and follow-up laboratory monitoring occurred in less than 20% of patients (16). Low rates of adherence to CAMESA-recommended laboratory monitoring were also observed in a retrospective study of 345 antipsychotic-treated children and youth referred to a psychopharmacology clinic at a children’s specialty hospital (11). This study additionally found no appreciable change in monitoring following the publication of CAMESA guidelines, with 35 and 39% of patients receiving any laboratory monitoring in the periods preceding and following publication (17). Another chart audit of 180 children and youth found that baseline and follow-up laboratory monitoring occurred in less than 50% of patients 5 years following the publication of CAMESA guidelines (18). In the only Canadian population-based study, the prevalence of baseline and follow-up (3 months to 1 year) metabolic monitoring in 6916 Alberta children and youth was 17 and 35%, respectively (19). Furthermore, most children (58%) did not undergo guideline-recommended testing during follow-up, and laboratory monitoring was less prevalent in treatment naïve children and youth (19).

Yet, despite these findings, additional research examining adherence to recommended laboratory monitoring in antipsychotic-treated children and youth is needed, for several reasons. First, most Canadian research is based on chart audits of small numbers of children and youth (16–18). Moreover, the lone Canadian population-based study was conducted just 3 years following the publication of the CAMESA guidelines (19). Population-based studies evaluating guideline adherence over a longer period of follow are needed to understand whether best monitoring practices have been adopted into clinical practice. Second, few population-based studies have explored whether disparities exist in the receipt of guideline-recommended laboratory monitoring. This is important because such disparities can result in a higher frequency of adverse health outcomes in specific sub-populations of patients. In one United States study using Medicaid claims data, the strongest determinants of glucose testing were the presence of multiple mental health co-morbidities and being hospitalized or seen in an emergency department, while having two or more medical office visits and serious mental illness were associated with lipid testing (20). Similar findings have been observed in more recent studies, with serious mental illness and greater contact with the health care system being determinants of metabolic monitoring (16, 21, 22). The setting in which care is being delivered has also been examined as a determinant of metabolic monitoring, with one study finding low adherence to monitoring guidelines across child psychiatry, pediatric and family practice clinics (23). However, this study explored relatively few sociodemographic and clinical correlates of testing, limiting insight into which individuals are at greatest risk for guidelines not being followed. Specifically, it is unknown if guideline adherence varies according to socioeconomic status, prescriber type, the concomitant receipt of psychotropic medications that may increase the risk of adverse effects with antipsychotics (e.g., stimulants, benzodiazepines, antidepressants) or clinical conditions in which the use of antipsychotics is typically off-label, such as autism spectrum disorder and attention deficit/hyperactivity disorder (ADHD). Moreover, studies from the United States examining adherence to monitoring guidelines were limited to children and youth with specific forms of insurance, and therefore excluded large groups of individuals lacking insurance and who may be most risk of poor monitoring (20, 24, 25). Studies including all children and youth regardless of insurance status, are needed to better understand disparities in guideline-recommended monitoring and inform the development of strategies promoting adherence to best practices.

Between January 2018 and March 2019, all children and youth aged 24 and under in Ontario, Canada received prescription medication at no cost through a publicly-funded universal pharmacare program (26). Prescription drug data were therefore available for all children and youth during this period. Because all required laboratory testing is covered by the publicly-funded health care system and these data are also available for the entire population, we conducted a study exploring variation in the receipt of CAMESA-recommended laboratory monitoring among all antipsychotic-treated children and youth in Ontario. Our main objectives were to determine the prevalence of guideline-recommended laboratory testing at baseline and at 3 and 6 months following the commencement of treatment. We also sought to identify correlates of baseline and follow-up recommended monitoring.

## Methods

### Setting

We conducted a population-based cohort study of all Ontario residents aged 0 to 24 who were newly dispensed an antipsychotic between April 1, 2018, and March 31, 2019. We selected an upper age limit of 24 to align with the United Nations definition of youth as individuals between the ages of 15 and 24.^1^

### Data sources

We used Ontario’s administrative health databases. These datasets were linked using unique encoded identifiers and analyzed at ICES (formerly known as the Institute for Clinical Evaluative Sciences). We identified claims for antipsychotics using the Ontario Drug Benefit database, which contains comprehensive records of all publicly-funded medications dispensed to Ontario residents. In addition, we identified prescriptions for stimulants and benzodiazepines using the Narcotics Monitoring System database, which contains comprehensive population records of all controlled-substances prescriptions dispensed from community pharmacies in Ontario, regardless of payer. We used the ICES Physician Database and Corporate Provider Database to determine prescriber specialty and the Registered Persons Database, a registry of all individuals eligible for the Ontario Health Insurance Plan (OHIP), to ascertain demographic characteristics for all children and youth dispensed antipsychotics over the study period. Specific demographic variables included age, sex, urban or rural residence, and neighborhood income quintile, derived by linking 2016 Census data to individual residential postal codes using the Postal Code Conversion File. To define patient comorbidity, we used the OHIP database to identify claims for physician services, and obtained diagnostic information from inpatient hospital admissions, emergency department visits and mental health-related hospitalizations using the Canadian Institute for Health Information’s Discharge Abstract Database, National Ambulatory Care Reporting System database and Ontario Mental Health Reporting System database, respectively. Specific mental health and neurodevelopmental conditions of interest included past diagnoses at any time prior to initiating an antipsychotic of schizophrenia or other psychotic disorders, ADHD, and autism spectrum disorder (see Supplementary Appendix for diagnostic codes). We also captured mental health hospitalizations and emergency department visits in the year preceding the initiation of antipsychotic therapy. We further categorized these encounters into diagnostic groups comprising substance use disorders, mood disorders, anxiety disorders, obsessive compulsive disorder, personality disorders and self-harm, based on the primary or main diagnosis (see Supplementary Appendix for codes). These definitions have been widely used for mental health system performance measurement in Canada (27–30). We identified individuals with pre-existing diabetes using the Ontario Diabetes Database, reasoning that these individuals may undergo more frequent laboratory monitoring for blood glucose.

To determine whether individuals prescribed antipsychotics received recommended laboratory monitoring, we used the OHIP database to identify claims for laboratory tests billed to the publicly funded health insurance plan and the Ontario Laboratory Information System (OLIS) database, an electronic repository containing information on provincial laboratory orders, patient demographics and provider information. OLIS includes data from hospital, commercial and public health laboratories, with 91% coverage of the province’s total annual laboratory testing as of 2016 (31). We excluded children and youth initiating antipsychotic treatment at the Hospital for Sick Children because this facility did not contribute data to OLIS during the study period. The use of data in this project was authorized under section 45 of Ontario’s Personal Health Information Protection Act, which does not require review by a Research Ethics Board.

### Study population and outcomes

We identified all individuals 0 to 24 years of age who were newly dispensed a prescription antipsychotic between April 1, 2018, and March 31, 2019, using the Ontario Drug Benefit database. Because the publicly-funded drug benefit program covers a maximum of 100 days’ supply of medication per prescription, we used a three-month look-back period to ascertain new use. We defined the prevalence of baseline laboratory monitoring as the number of individuals receiving at least one recommended laboratory test between 30 days before and 14 days after the first dispensing of a prescription antipsychotic divided by the total number of individuals newly dispensed an antipsychotic over the accrual period.

To examine the prevalence of follow-up testing 3 and 6 months after initiating antipsychotic therapy, we restricted our cohort to individuals newly dispensed a prescription antipsychotic between April 1, 2018, and September 30, 2018. We next defined a period of ongoing antipsychotic use as the dispensing of a prescription antipsychotic within 1.5 times the previous prescription days’ supply to allow a grace period between repeat prescriptions. This is a commonly used approach for preventing person-time on continuous therapy from being misclassified as unexposed in cases where individuals do not fill their prescription at precise intervals defined by the amount of medication dispensed or if the calculated or pharmacist-recorded days’ supply is too short (32).We censored individuals at the time of treatment discontinuation (no refills within 1.5 times the previous prescription days’ supply), turning 25 years old, or the end of follow-up (March 31, 2019), whichever occurred first. We did not censor individuals who switched between antipsychotics. We determined the prevalence of guideline-recommended testing at approximately 3 and 6 months after antipsychotic therapy initiation, defined as the number of individuals receiving at least one guideline-recommended laboratory test 60 to 120 days and 150 to 200 days following the first dispensing of an antipsychotic divided by the number of individuals still receiving ongoing treatment in those follow-up windows. The specific laboratory tests examined in all analyzes were glucose, serum transaminases, prolactin, total cholesterol, high-density lipoprotein (HDL) and low-density lipoprotein (LDL) cholesterol, triglycerides, and other lipids.

### Statistical analysis

We summarized baseline characteristics of children and youth initiating antipsychotics using counts and percentages for categorical variables and medians with interquartile ranges (IQR) for continuous variables. We determined the prevalence of baseline and follow-up testing for the overall sample and according to baseline demographic variables (i.e., sex, age-category, urban or rural residence, neighborhood income quintile), clinical and health system variables (i.e., comorbidity status, mental health-related hospital admissions or emergency department visits in the year preceding antipsychotic initiation, receipt of benzodiazepines, stimulants or antidepressants), and antipsychotic prescriber specialty (i.e., family physician, pediatrician, child and adolescent psychiatrist or developmental pediatrician, other). We ascertained individual comorbidity in the prior 2 years using the Johns Hopkins ACG® System Version 10 case-mix assignment software (33). We specifically used ACG® System Aggregated Diagnosis Groups (ADGs), which are clusters of diagnostic codes with similar severity and expected persistence. The count of ADGs ranges from 0 to 32, with higher values reflecting more comorbidity. We categorized comorbidity status as 0 to 5, 6 to 9 and ≥ 10 ADGs.

We used multivariable log-Poisson regression models with robust variance estimators to quantify the associations between demographic, clinical and health systems variables with the receipt of baseline and follow-up laboratory monitoring (34, 35). We expressed results as prevalence ratios (PRs) and 95% confidence intervals (CI).

## Results

We identified 27,718 children and youth who were newly dispensed an antipsychotic medication between April 1, 2018, and March 31, 2019. Most (*n* = 27,222; 98.2%) received a second-generation antipsychotic. The median duration of continuous use was 7.6 weeks (interquartile range 4.3 to 18.1 weeks). A slight majority (*n* = 14,186; 51.2%) were female, and the median age was 19 years (IQR 15 to 21), with 9.4% being younger than 10 (*n* = 2,591). About one-third of the cohort (*n* = 9,382; 33.9%) had a mental health-related hospitalization or emergency department visit in the year before receiving an antipsychotic (Table 1). In addition, 3,827 (13.8%), 7,498 (27.1%) and 2,887 (10.4%) individuals had healthcare encounters for schizophrenia, ADHD and autism spectrum disorder, respectively, at any time before receiving an antipsychotic. Overall, 7,363 (27.6%) underwent laboratory testing at least once during the baseline, 3- and 6-month follow-up periods, with 64 (0.23%) individuals being monitored at all three time points.

**Table 1:** Characteristics of children and youth newly dispensed an antipsychotic between April 1, 2018, and March 31, 2019.

Characteristics	*N* = 27,718
Age (median, IQR)	19 (15 to 21)
0 to 9	2,591 (9.4%)
10 to 14	3,384 (12.2%)
15 to 19	9,596 (34.6%)
20 to 24	12,147 (43.8%)
Sex	
Female	14,186 (51.2%)
Male	13,532 (48.8%)
Neighborhood income quintile	
1 (lowest)	6,981 (25.2%)
2	5,735 (20.7%)
3	5,201 (18.8%)
4	4,805 (17.3%)
5 (highest)	4,996 (18.0%)
Urban or rural residence	
Urban	24,483 (88.3%)
Rural	3,235 (11.7%)
Diabetes diagnosis	394 (1.4%)
John’s Hopkins Aggregated Diagnosis Groups	
0 to 5	12,502 (45.1%)
6 to 9	10,703 (38.6%)
≥ 10	4,513 (16.3%)
Emergency department visit or hospitalization for mental health diagnoses (previous year)	
Any^a^	9,382 (33.9%)
Anxiety disorders^b^	2,000 (7.2%)
Deliberate self-harm^c^	1,280 (4.6%)
Mood disorder^d^	3,546 (12.8%)
Obsessive compulsive disorder or related disorders^e^	91 (0.3%)
Personality disorders^f^	631 (2.3%)
Schizophrenia and other psychotic disorders^g^	1,541 (5.6%)
Substance-related and addictivedisorders^h^	1,678 (6.1%)
Trauma/stressor related disorder^i^	2,215 (8.0%)
Other	1,036 (3.7%)
Healthcare encounter for mental health condition (any time prior to index date)	
Schizophrenia and other psychotic disorders	3,827 (13.8%)
ADHD	7,498 (27.1%)
ASD	2,887 (10.4%)
Prescribed medications	
Stimulant	3,117 (11.3%)
Benzodiazepine	1,093 (3.9%)
Antidepressant	7,957 (28.7%)
Prescriber type	
Family medicine	10,401 (37.5%)
Psychiatrist or developmental pediatrician	11,251 (40.6%)
Pediatrician	3,735 (13.5%)
Other	2,331 (8.4%)

^a^Broad category that includes anxiety disorders, mood disorders, obsessive compulsive or related disorders, personality disorders, schizophrenia and other psychotic disorders, substance use disorders, trauma/stressor related disorder, and other disorders.

^b^Includes organic anxiety disorder, phobic anxiety disorders, other anxiety disorders, emotional disorders with onset specific to childhood and elective mutism.

^c^Includes intentional self-poisonings by alcohol or other drugs, chemicals, gases/vapors or pesticides and intentional self-harm (e.g., submersion, firearm discharge, sharp object).

^d^Includes organic mood disorders, manic episode, bipolar affective disorder, depressive episode, recurrent depressive disorder, persistent mood [affective] disorders, other mood [affective] disorders, unspecified mood [affective] disorders and mild mental and behavioral disorders associated with the puerperium, not elsewhere classified.

^e^Includes obsessive–compulsive disorder, hypochondriacal disorder and trichotillomania.

^f^Includes organic personality disorder, schizotypal disorder, specific personality disorders, mixed and other personality disorders, enduring personality changes, not attributable to brain damage and disease, other disorders of adult personality and behavior and unspecified disorder of adult personality and behavior.

^g^Includes other mental disorders due to brain damage and dysfunction and to physical disease, schizophrenia, persistent delusional disorders, acute and transient psychotic disorders, induced delusional disorder, schizoaffective disorders, other nonorganic psychotic disorders, unspecified nonorganic psychosis and severe mental and behavioral disorders associated with the puerperium, not elsewhere classified.

^h^Includes mental and behavioral disorders due to use of alcohol, opioids, cannabinoids, sedative/hypnotics, cocaine, other stimulants (including caffeine), hallucinogens, tobacco, volatile solvents or other psychoactive substances. Also includes abuse of non-dependence-producing substances and pathological gambling.

^i^Includes reaction to severe stress and adjustment disorders, reactive attachment disorder of childhood and disinhibited attachment disorder of childhood. See Supplementary Appendix for exact codes.

### Baseline monitoring

Overall, 6,505 (23.5%) antipsychotic-treated children and youth received at least one guideline-recommended baseline test. Monitoring was more prevalent in children treated with first-generation antipsychotics (211/496; 42.5%) than second-generation antipsychotics (6,294/27,222; 23.1%). Blood glucose and serum transaminase testing were most common, received by 5,870 (21.2%) and 4,706 (17.0%) of children and youth, with lipid (*n* = 2,180; 7.9%) and prolactin (*n* = 720; 2.6%) testing undertaken less frequently. Following multivariable adjustment, baseline monitoring was more prevalent among those aged 10 to 14 years (PR 1.20; 95% CI 1.04 to 1.38), 15 to 19 years (PR 1.60; 95% CI 1.41 to 1.82) and 20 to 24 years (PR 1.71; 95% CI 1.50 to 1.94) relative to individuals younger than 10 (Table 2). The frequency of baseline monitoring was slightly greater in the highest-income relative to the lowest-income neighborhoods (25.1% vs. 23.4%; PR 1.09; 95% CI 1.02 to 1.16). In addition, the prevalence of monitoring was higher in children and youth with any mental health-related hospitalizations or emergency department visits in the year prior to initiating therapy (37.4% vs. 16.3%; PR 1.76; 95% CI 1.65 to 1.87), those with a past diagnosis of schizophrenia or psychotic disorder (37.6% vs. 21.2%; PR 1.20; 95% CI 1.14 to 1.26), individuals with pre-existing diabetes (36.3% vs. 23.3%; PR 1.35; 95% CI 1.19 to 1.54) and individuals who were co-prescribed benzodiazepines (32.0% vs. 23.1%; PR 1.13; 95% CI 1.04 to 1.24). Baseline monitoring was also more frequent among children and youth whose treatment was initiated by a child and adolescent psychiatrist or developmental pediatrician relative to a family physician (31.4% vs. 18.2%; PR 1.41, 95% CI 1.34 to 1.48). Baseline monitoring was also more prevalent among those with 6 to 9 ADGs (PR 1.41; 95% CI 1.34 to 1.48) relative to those with 5 or fewer ADGs. Conversely, monitoring was less common in individuals with a diagnosis of ADHD (19.4% vs. 25.0%; PR 0.86; 95% CI 0.81 to 0.90) and autism spectrum disorder (16.3% vs. 24.3%; PR 0.89, 95% CI 0.82 to 0.97), as well as those co-prescribed stimulants (13.7% vs. 24.7%; PR 0.83; 95% CI 0.75 to 0.91) or antidepressants (22.8% vs. 23.7%; PR 0.89; 95% CI 0.85 to 0.93). Although baseline monitoring was more frequent among females relative to males (24.6% vs. 22.3%), females were less likely to undergo baseline monitoring following multivariable analysis (PR 0.95; 95% CI 0.91 to 0.99) (Table 2).

**Table 2:** Prevalence ratios for receipt of any baseline monitoring test among children and youth newly dispensed an antipsychotic between April 1, 2018 and March 31, 2019.

Variable	Number (%) with a baseline test	Unadjusted prevalence ratio (95% CI)	Adjusted prevalence ratio (95% CI)
Age			
0 to 9 (ref)	271/2591 (10.5%)	1.00	1.00
10 to 14	507/3384 (15.0%)	1.43 (1.25–1.64)	1.20 (1.04–1.38)
15 to 19	2471/9596 (25.8%)	2.46 (2.19–2.77)	1.60 (1.41–1.82)
20 to 24	3256/12147 (26.8%)	2.56 (2.28–2.88)	1.71 (1.50–1.94)
Sex			
Male (ref)	3023/13532 (22.3%)	1.00	1.00
Female	3482/14186 (24.6%)	1.10 (1.05–1.15)	0.95 (0.91–0.99)
Urban or rural residence
Urban (ref)	5760/24483 (23.5%)	1.00	1.00
Rural	745/3235 (23.0%)	0.98 (0.92–1.05)	1.08 (1.01–1.15)
Neighborhood income quintile			
1 (ref)	1633/6981 (23.4%)	1.00	1.00
2	1288/5735 (22.5%)	0.96 (0.90–1.02)	0.98 (0.92–1.04)
3	1191/5201 (22.9%)	0.98 (0.92–1.05)	1.00 (0.94–1.07)
4	1138/4805 (23.7%)	1.01 (0.95–1.08)	1.05 (0.99–1.12)
5	1255/4996 (25.1%)	1.07 (1.01–1.14)	1.09 (1.02–1.16)
John’s Hopkins Aggregated Diagnosis Groups			
0–5 (ref)	2333/12502 (18.7%)	1.00	1.00
6–9	2693/10703 (25.2%)	1.35 (1.28–1.42)	1.41 (1.34–1.48)
≥ 10	1479/4513 (32.8%)	1.76 (1.66–1.86)	1.07 (0.96–1.19)
Diabetes diagnosis			
No (ref)	6362/27324 (23.3%)	1.00	1.00
Yes	143/394 (36.3%)	1.56 (1.37–1.78)	1.35 (1.19–1.54)
Emergency department visit or hospitalization for mental health diagnoses (previous year)			
Any			
No (ref)	2995/18336 (16.3%)	1.00	1.00
Yes	3510/9382 (37.4%)	2.29 (2.20–2.39)	1.76 (1.65–1.87)
Anxiety disorders			
No (ref)	5800/25718 (22.6%)	1.00	1.00
Yes	705/2000 (35.3%)	1.56 (1.47–1.67)	0.97 (0.90–1.03)
Deliberate self-harm			
No (ref)	5980/26438 (22.6%)	1.00	1.00
Yes	525/1280 (41.0%)	1.81 (1.69–1.94)	1.10 (1.02–1.18)
Mood disorders			
No (ref)	5191/24172 (21.5%)	1.00	1.00
Yes	1314/3546 (37.1%)	1.73 (1.64–1.81)	0.97 (0.92–1.03)
Obsessive compulsive or related disorders			
No (ref)	6472/27627 (23.4%)	1.00	1.00
Yes	33/91 (36.3%)	1.55 (1.18–2.03)	0.97 (0.74–1.27)
Personality disorders			
No (ref)	6253/27087 (23.1%)	1.00	1.00
Yes	252/631 (39.9%)	1.73 (1.57–1.91)	1.00 (0.91–1.11)
Substance-related and addictive disorders			
No (ref)	5753/26040 (22.1%)	1.00	1.00
Yes	752/1678 (44.8%)	2.03 (1.91–2.15)	1.12 (1.05–1.19)
Trauma/stressor related disorder			
No (ref)	5767/25503 (22.6%)	1.00	1.00
Yes	738/2215 (33.3%)	1.47 (1.38–1.57)	0.90 (0.84–0.96)
Other
No (ref)	6139/26682 (23.0%)	1.00	1.00
Yes	366/1036 (35.3%)	1.54 (1.41–1.67)	1.09 (1.00–1.19)
Healthcare encounter for mental health diagnoses (any time prior to index date)			
Schizophrenia and other psychotic disorders			
No (ref)	5067/23891 (21.2%)	1.00	1.00
Yes	1438/3827 (37.6%)	1.77 (1.69–1.86)	1.20 (1.14–1.26)
ADHD			
No (ref)	5050/20220 (25.0%)	1.00	1.00
Yes	1455/7498 (19.4%)	0.78 (0.74–0.82)	0.86 (0.81–0.90)
ASD			
No (ref)	6035/24831 (24.3%)	1.00	1.00
Yes	470/2887 (16.3%)	0.67 (0.61–0.73)	0.89 (0.82–0.97)
Prescribed medication			
Stimulant			
No (ref)	6077/24601 (24.7%)	1.00	1.00
Yes	428/3117 (13.7%)	0.56 (0.51–0.61)	0.83 (0.75–0.91)
Benzodiazepine			
No (ref)	6155/26625 (23.1%)	1.00	1.00
Yes	350/1093 (32.0%)	1.39 (1.27–1.51)	1.13 (1.04–1.24)
Antidepressant			
No (ref)	4691/19761 (23.7%)	1.00	1.00
Yes	1814/7957 (22.8%)	0.96 (0.92–1.01)	0.89 (0.85 to 0.93)
Prescriber type			
Family Medicine (ref)	1894/10401 (18.2%)	1.00	1.00
Psychiatrist or developmental pediatrician	3527/11251 (31.4%)	1.72 (1.64–1.81)	1.41 (1.34–1.48)
Pediatrician	434/3735 (11.6%)	0.64 (0.58–0.70)	1.07 (0.96–1.19)
Other	650/2331 (27.9%)	1.53 (1.42–1.65)	1.38 (1.28–1.49)

### Follow-up monitoring

The prevalence of any follow-up laboratory testing at 3 months and 6 months among children and youth receiving continuous antipsychotic therapy at these time points was 13.0% (1,179 of 9,080) and 11.4% (597 of 5,261), respectively. Follow-up monitoring was more frequent in individuals receiving first generation antipsychotics, with 24.8% (28 of 113) and 21.5% (14 of 65) children and youth receiving continuous treatment at 3- and 6-months receiving any follow-up monitoring. Respective figures for children and youth receiving second generation antipsychotics were 12.8% (1,151 of 8,967) and 11.2% (583 of 5,196). Similar to baseline testing, the receipt of any 3- or 6-month follow-up testing was higher among older children and youth, those with prior diagnoses of schizophrenia or diabetes, those with a higher ADG comorbidity score, individuals co-prescribed benzodiazepines, and those with prescriptions written by a child and adolescent psychiatrist or developmental pediatrician (Table 3). Females were more likely than males to undergo 3-month (16.1% vs. 10.1%; PR 1.26, 95% CI 1.11 to 1.42) and 6-month follow-up testing (14.1% vs. 9.1%; PR 1.27, 95% CI 1.07 to 1.51).

**Table 3:** Prevalence ratios for receipt of any 3- and 6-month laboratory monitoring among children and youth newly dispensed an antipsychotic between April 1, 2018, and March 31, 2019.

Variable	3-month follow-up testing			6-month follow-up testing			Number (%)	Unadjusted prevalence ratio (95% CI)	Adjusted prevalence ratio (95% CI)	Number (%)	Unadjusted prevalence ratio (95% CI)	Adjusted prevalence ratio (95% CI)
Age						
0 to 9 (ref)	59/985 (6.0%)	1.00	1.00	44/676 (6.5%)	1.00	1.00
10 to 14	121/1413 (8.6%)	1.43 (1.06–1.93)	1.23 (0.91–1.67)	71/909 (7.8%)	1.20 (0.84–1.72)	1.04 (0.71–1.51)
15 to 19	441/2935 (15.0%)	2.51 (1.93–3.26)	1.73 (1.29–2.31)	218/1636 (13.3%)	2.05 (1.50–2.79)	1.32 (0.92–1.90)
20 to 24	558/3747 (14.9%)	2.49 (1.92–3.22)	1.82 (1.36–2.45)	264/2040 (12.9%)	1.99 (1.46–2.70)	1.33 (0.92–1.92)
Sex						
Male (ref)	483/4769 (10.1%)	1.00	1.00	261/2874 (9.1%)	1.00	1.00
Female	696/4311 (16.1%)	1.59 (1.43–1.78)	1.26 (1.11–1.42)	336/2387 (14.1%)	1.55 (1.33–1.81)	1.27 (1.07–1.51)
Urban or rural residence						
Urban (ref)	1029/7960 (12.9%)	1.00	1.00	533/4601 (11.6%)	1.00	1.00
Rural	150/1120 (13.4%)	1.04 (0.88–1.22)	1.21 (1.03–1.41)	64/660 (9.7%)	0.84 (0.65–1.07)	0.96 (0.75–1.22)
Neighborhood income quintile						
1 (ref)	270/2192 (12.3%)	1.00	1.00	133/1237 (10.8%)	1.00	1.00
2	258/1880 (13.7%)	1.11 (0.95–1.31)	1.09 (0.94–1.28)	123/1080 (11.4%)	1.06 (0.84–1.33)	1.04 (0.83–1.31)
3	186/1690 (11.0%)	0.89 (0.75–1.07)	0.89 (0.75–1.06)	102/1000 (10.2%)	0.95 (0.74–1.21)	0.96 (0.76–1.23)
4	234/1638 (14.3%)	1.16 (0.99–1.37)	1.13 (0.96–1.32)	121/964 (12.6%)	1.17 (0.93–1.47)	1.13 (0.90–1.42)
5	231/1680 (13.8%)	1.12 (0.95–1.31)	1.08 (0.92–1.27)	118/980 (12.0%)	1.12 (0.89–1.41)	1.09 (0.86–1.38)
John’s Hopkins Aggregated Diagnosis Groups						
0–5 (ref)	381/4341 (8.8%)	1.00	1.00	207/2542 (8.1%)	1.00	1.00
6–9	490/3426 (14.3%)	1.63 (1.44–1.85)	1.42 (1.25–1.61)	255/2010 (12.7%)	1.56 (1.31–1.85)	1.23 (1.02–1.48)
≥ 10	308/1313 (23.5%)	2.67 (2.33–3.06)	1.15 (0.93–1.42)	135/709 (19.0%)	2.34 (1.91–2.86)	1.04 (0.77–1.41)
Diabetes diagnosis						
No (ref)	1142/8945 (12.8%)	1.00	1.00	577/5177 (11.1%)	1.00	1.00
Yes	37/135 (27.4%)	2.15 (1.62–2.84)	1.72 (1.30–2.27)	20/84 (23.8%)	2.14 (1.45–3.16)	1.67 (1.12–2.47)
Emergency department visit or hospitalization for mental health diagnoses (previous year)						
Any						
No (ref)	692/6473 (10.7%)	1.00	1.00	354/3846 (9.2%)	1.00	1.00
Yes	487/2607 (18.7%)	1.75 (1.57–1.94)	1.07 (0.90–1.27)	243/1415 (17.2%)	1.87 (1.60–2.17)	1.23 (0.96–1.59)
Anxiety disorders						
No (ref)	1066/8565 (12.4%)	1.00	1.00	548/4977 (11.0%)	1.00	1.00
Yes	113/515 (21.9%)	1.76 (1.48–2.09)	1.22 (1.01–1.47)	49/284 (17.3%)	1.57 (1.20–2.05)	1.02 (0.76–1.36)
Deliberate self-harm						
No (ref)	1096/8729 (12.6%)	1.00	1.00	559/5071 (11.0%)	1.00	1.00
Yes	83/351 (23.6%)	1.88 (1.55–2.29)	1.12 (0.91–1.38)	38/190 (20.0%)	1.81 (1.35–2.44)	1.03 (0.75–1.42)
Mood disorders						
No (ref)	987/8086 (12.2%)	1.00	1.00	502/4736 (10.6%)	1.00	1.00
Yes	192/994 (19.3%)	1.58 (1.38–1.82)	1.00 (0.84–1.19)	95/525 (18.1%)	1.71 (1.40–2.08)	1.02 (0.79–1.30)
Obsessive compulsive or related disorders						
No (ref)	1169/9053 (12.9%)	1.00	1.00	NA	1.00	1.00
Yes	10/27 (37.0%)	2.87 (1.75–4.70)	1.83 (1.14–2.95)	NA	1.77 (0.64–4.87)	0.94 (0.34–2.56)
Personality disorders						
No (ref)	1135/8898 (12.8%)	1.00	1.00	579/5168 (11.2%)	1.00	1.00
Yes	44/182 (24.2%)	1.90 (1.46–2.47)	1.09 (0.83–1.44)	18/93 (19.4%)	1.73 (1.13–2.63)	0.95 (0.62–1.45)
Substance-related and addictive disorders						
No (ref)	1095/8676 (12.6%)	1.00	1.00	557/5054 (11.0%)	1.00	1.00
Yes	84/404 (20.8%)	1.65 (1.35–2.01)	1.09 (0.88–1.35)	40/207 (19.3%)	1.75 (1.31–2.34)	1.12 (0.82–1.52)
Trauma/stressor related disorder						
No (ref)	1059/8480 (12.5%)	1.00	1.00	534/4930 (10.8%)	1.00	1.00
Yes	120/600 (20.0%)	1.60 (1.35–1.90)	1.07 (0.88–1.28)	63/331 (19.0%)	1.76 (1.39–2.23)	1.11 (0.85–1.44)
Other						
No (ref)	1113/8736 (12.7%)	1.00	1.00	560/5048 (11.1%)	1.00	1.00
Yes	66/344 (19.2%)	1.51 (1.20–1.88)	1.20 (0.95–1.52)	37/213 (17.4%)	1.57 (1.16–2.12)	1.21 (0.87–1.68)
Healthcare encounter for mental health diagnoses (any time prior to index date)						
Schizophrenia and other psychotic disorders						
No (ref)	945/7721 (12.2%)	1.00	1.00	461/4429 (10.4%)	1.00	1.00
Yes	234/1359 (17.2%)	1.41 (1.23–1.60)	1.16 (1.01–1.34)	136/832 (16.4%)	1.57 (1.32–1.87)	1.32 (1.09–1.61)
ADHD						
No (ref)	861/6277 (13.7%)	1.00	1.00	453/3585 (12.6%)	1.00	1.00
Yes	318/2803 (11.3%)	0.83 (0.73–0.93)	0.99 (0.88–1.13)	144/1676 (8.6%)	0.68 (0.57–0.81)	0.79 (0.66–0.96)
ASD						
No (ref)	1035/7721 (13.4%)	1.00	1.00	501/4301 (11.7%)	1.00	1.00
Yes	144/1359 (10.6%)	0.79 (0.67–0.93)	1.08 (0.91–1.28)	96/960 (10.0%)	0.86 (0.70–1.06)	1.15 (0.92–1.43)
Prescribed medication						
Stimulant						
No (ref)	1073/7773 (13.8%)	1.00	1.00	532/4369 (12.2%)	1.00	1.00
Yes	106/1307 (8.1%)	0.59 (0.49–0.71)	0.80 (0.66–0.98)	65/892 (7.3%)	0.60 (0.47–0.77)	0.87 (0.67–1.14)
Benzodiazepine						
No (ref)	1099/8758 (12.6%)	1.00	1.00	558/5070 (11.0%)	1.00	1.00
Yes	80/322 (24.8%)	1.98 (1.62–2.41)	1.38 (1.13–1.69)	39/191 (20.4%)	1.86 (1.39–2.48)	1.32 (0.98–1.77)
Antidepressant						
No (ref)	809/6575 (12.3%)	1.00	1.00	395/3771 (10.5%)	1.00	1.00
Yes	370/2505 (14.8%)	1.20 (1.07–1.35)	1.00 (0.89–1.12)	202/1490 (13.6%)	1.29 (1.10–1.52)	1.10 (0.93–1.30)
Prescriber type						
Family medicine (ref)	395/3530 (11.2%)	1.00	1.00	201/1955 (10.3%)	1.00	1.00
Psychiatrist or developmental pediatrician	561/3221 (17.4%)	1.56 (1.38–1.75)	1.42 (1.25–1.61)	273/1827 (14.9%)	1.45 (1.23–1.72)	1.23 (1.02–1.48)
Pediatrician	131/1720 (7.6%)	0.68 (0.56–0.82)	1.15 (0.93–1.42)	82/1143 (7.2%)	0.70 (0.55–0.89)	1.04 (0.77–1.41)
Other	92/609 (15.1%)	1.35 (1.09–1.67)	1.37 (1.11–1.68)	41/336 (12.2%)	1.19 (0.87–1.63)	1.16 (0.85–1.60)

## Discussion

In our population-based study of children and youth initiating antipsychotic therapy, we found that fewer than one in four children and youth initiating antipsychotic therapy received any baseline laboratory monitoring. Follow-up monitoring was also infrequent, with only 13.0 and 11.4% of individuals receiving 3- and 6-month testing, respectively. Baseline and follow-up monitoring were more common among older recipients, those co-prescribed benzodiazepines, those with a past diagnosis of diabetes or schizophrenia and other psychotic disorders, individuals with a mental-health related hospitalization or emergency department visit in the year prior to commencing therapy, and those prescribed antipsychotics by a child and adolescent psychiatrist or developmental pediatrician. However, monitoring was infrequent even in these patients. Overall, our findings suggest that best laboratory monitoring practices for children and youth receiving antipsychotics are occurring infrequently 7 years following the publication of the CAMESA guidelines.

Our study has important implications for clinical practice. The potential for iatrogenic harm with antipsychotics in children and youth is well documented, with metabolic abnormalities such as dyslipidemia, hyperglycemia and obesity occurring early during treatment and imparting a 2- to 3-fold increase in risk for type 2 diabetes relative to children and youth who are not treated with these drugs (1–4). This risk increases with higher cumulative doses, longer treatment duration, and adjunctive antidepressant use, and remains elevated after discontinuation (3, 4, 36). Moreover, adverse metabolic effects developing in childhood may increase the risk of early and long-term cardiovascular disease (6–8). In light of the potential for serious adverse effects and the low prevalence of monitoring in all children and youth, tailored interventions are needed to promote best monitoring practices when antipsychotics are prescribed. This assertion is supported by past studies from Canada and the United States finding small changes in monitoring before and after the publication of clinical guidelines (17, 37, 38). Furthermore, changes in monitoring practices observed in studies of quality improvement educational initiatives targeting prescribers have been modest, with a systematic review of six studies finding median postintervention glucose, lipid, and waist circumference screening rates of 39, 37 and 16%, respectively (39). These findings suggest that the publication of guidelines and interventions targeting clinician knowledge and practice enhancements are insufficient for improving metabolic screening rates in antipsychotic-treated children and youth.

Our data do not allow us to conclusively determine whether low guideline adherence reflects a lack of awareness among clinicians and families for the need for regular monitoring or challenges obtaining blood work at regular intervals among families overburdened by multiple appointments and competing needs for children and youth with mental health and neurodevelopmental conditions (40–43).

Patient comfort and education regarding the need for regular blood work may represent possible individual-level barriers to adopting metabolic monitoring recommendations in routine clinical practice. This is consistent with our findings that past diagnoses of diabetes and schizophrenia and prior mental health-related hospitalizations and emergency department visits were associated with a higher prevalence of baseline and follow-up monitoring, as these individuals may be more comfortable with or aware of the need for routine monitoring involving routine blood work. Our finding that laboratory monitoring was associated with increasing age may also reflect greater comfort with regular blood work in adolescents and youth, as past research has found higher levels of distress with venipuncture among younger children relative to adolescents (44). Similarly, anxiety related to medical procedures among children with neurodevelopmental disorders such as ADHD and autism spectrum disorder may explain the lower monitoring prevalence in these children and youth (45, 46). Toolkits developed to support obtaining blood work from children and youth with neurodevelopmental disorders may help facilitate routine metabolic monitoring when antipsychotics are prescribed for children and youth with neurodevelopmental disorders (47). Similarly, patient-centered programs offering flexible laboratory appointment times, blood work conducted in the patient’s home rather than an office setting, and additional support to promote patient comfort may help overcome anxiety and fear associated with specimen collection, although research is required to ascertain their effectiveness in promoting regular monitoring (46, 48). The higher prevalence of baseline and follow-up monitoring among individuals receiving benzodiazepines may reflect the short-term use of these drugs for procedural anxiolysis. Although this practice has been found to be generally well tolerated, studies of prophylactic benzodiazepines in children have generally focused on operative and diagnostic procedures, and the efficacy and safety for reducing anxiety related to blood work is unknown (49). Our finding of a slightly higher prevalence of baseline monitoring in children and youth living in the highest relative to the lowest income neighborhoods suggests that structural interventions addressing barriers such as the costs of attending separate medical and laboratory appointments and time away from work may be needed to support low-income families in obtaining recommended monitoring.

Provider-level barriers may also contribute to low rates of recommended monitoring, with non-specialist physicians being reluctant to undertake metabolic monitoring without access to expert guidance on the management of abnormalities. This is consistent with our finding of a higher screening prevalence among children and youth seen by child and adolescent psychiatrists and developmental pediatricians compared to primary care providers and past research showing a general awareness of and agreement with the need for metabolic monitoring among psychiatrists (50–52). A similar finding has been observed in a United States study, in that the odds of receiving metabolic monitoring were 42% higher in children and youth receiving shared care including a mental health provider relative to treatment from a primary care provider alone (53). Shared care models, including telemedical health programs connecting primary care providers to pediatric mental health specialists, represent a promising mechanism for optimizing antipsychotic prescribing and monitoring in children and youth (54, 55). Training clinicians to provide specialist-level care to children and youth with mental health and neurodevelopmental conditions is another approach for promoting best practices in antipsychotic laboratory monitoring of children and youth. This approach has been implemented in Ontario through Project Extension of Community Health Outcomes (ECHO) (56, 57). Project ECHO Ontario uses a continuing professional education model to train health care providers throughout Ontario to provide specialist-level care to children and youth with mental health and neurodevelopmental conditions. However, whether such initiatives can improve adherence to metabolic monitoring in antipsychotic-treated children and youth remains unknown, with further evaluation needed. In addition, further research is needed to investigate the role of health system- and organizational-level characteristics (e.g., presence of laboratory facilities onsite) and interventions addressing such barriers on adherence to monitoring guidelines.

Our study has some limitations. First, we could not ascertain the prevalence of monitoring for other guideline-recommended parameters, such as blood pressure, body mass index and waist circumference. Past research suggests that adherence to these monitoring parameters is higher than that for those requiring routine blood work (51, 52). It is therefore possible that clinicians may be undertaking metabolic monitoring only in those children demonstrating changes in anthropomorphic measures. However, even if this were the case, the prevalence of metabolic monitoring remains inadequate given that most antipsychotic-treated children and youth experience weight gain or obesity within 12 weeks of initiating treatment (2). Second, we could not determine the indication for antipsychotic use. Prior Canadian research indicates that these drugs are generally used off-label for children and youth with ADHD, conduct disorders and mood disorders (19, 58). Third, diagnostic categories of mental health and neurodevelopmental conditions were derived using administrative algorithms published in earlier studies and reports, and were not validated for use in this study (27, 59). Fourth, we could not account for the dose of antipsychotic in our analyzes. Fifth, we could not ascertain whether glucose and lipid testing were performed under fasting conditions. Finally, our study was conducted in a single Canadian province in the year when universal funding of medications for all children and youth was introduced, potentially limiting the generalizability of our findings. However, our study includes all children and youth newly initiating antipsychotic drugs in a setting of publicly funded healthcare and pharmacare. Our findings suggest that barriers unrelated to health insurance status continue to undermine the uptake of guideline-recommended laboratory monitoring in antipsychotic-treated children and youth and may be transferable to similar settings where prescription drugs and routine healthcare are provided at no cost to children and youth.

In conclusion, most children and youth initiating antipsychotic therapy do not undergo guideline-recommended baseline and follow-up metabolic monitoring. Further research clarifying reasons for poor guideline uptake is needed to inform the development and evaluation of interventions that optimize laboratory metabolic monitoring in children and youth treated with antipsychotics. In addition, research examining the role of alternative healthcare delivery models, such as telehealth and programs training primary care physicians to provide specialist care, prophylactic procedural anxiolysis and toolkits to assist with blood draws is warranted to determine whether such interventions can improve rates of metabolic monitoring.

## Data availability statement

The data analyzed in this study is subject to the following licenses/restrictions: The datasets generated and/or analyzed during the current study are not publicly available due to data sharing agreements which prohibit ICES from making the data set publicly available. The data set from this study is held securely in coded form at ICES, and access may be granted to those who meet pre-specified criteria for confidential access, available at https://www.ices.on.ca/DAS,understanding that the programs may rely upon coding templates or macros that are unique to ICES. Requests to access these datasets should be directed to https://www.ices.on.ca/DAS.

## Ethics statement

Ethical review and approval was not required for the study on human participants in accordance with the local legislation and institutional requirements. Written informed consent from the participants’ legal guardian/next of kin was not required to participate in this study in accordance with the legislation and the institutional requirements.

## Author contributions

TA, TW, TG, MP, MT, KP, WG, MM, YL, and DJ conceptualized and designed the study, and were involved in the interpretation of the data. TW conducted the analyzes. TA drafted the initial manuscript. All authors critically reviewed the manuscript for intellectual content, revised the manuscript, and approved the final manuscript as submitted and agree to be accountable for all aspects of the work in ensuring that questions related to the accuracy or integrity of any part of the work are appropriately investigated and resolved.

## Funding

This study was funded by the Canadian Institutes of Health Research (funding reference number 166149).

## Conflict of interest

MP has received consulting fees for unrelated work from Addis & Associates/Roche and the Government of Nova Scotia. MT has received consulting fees for unrelated work from Green Shield Canada and the Canadian Agency for Drugs and Technologies in Health. TG has received funding from the Ontario MOH and the Canadian Agency for Drugs and Technologies in Health for unrelated work.

The remaining authors declare that the research was conducted in the absence of any commercial or financial relationships that could be construed as a potential conflict of interest.

## Publisher’s note

All claims expressed in this article are solely those of the authors and do not necessarily represent those of their affiliated organizations, or those of the publisher, the editors and the reviewers. Any product that may be evaluated in this article, or claim that may be made by its manufacturer, is not guaranteed or endorsed by the publisher.

## Footnotes

^1^ https://www.un.org/en/global-issues/youth
