# Supplementary material for: Adherence to antipsychotic laboratory monitoring guidelines in children and youth: a population-based study
Source: Front Psychiatry. 2023 May 12;14:1172559. doi: 10.3389/fpsyt.2023.1172559 (PMC10217777; doi:10.3389/fpsyt.2023.1172559)
Supplement: Supplementary file 1 [file Table_1.docx]

**Supplemental Appendix: Mental Health Diagnoses**

| **Clinical category** | **ICD-9-CM codes (OMHRS DSM 5)^a^** | **ICD-10-CA codes (DAD/NACRS)^a^** |
| --- | --- | --- |
| **Any mental health and addictions** | Any OMHRS record (including missing, except for 290.x, 294.x in primary diagnosis). Excluded if primary diagnosis missing and provisional=17. | F06-F99, X60-X84, Y10-Y19, Y28 when the primary diagnosis is not F06-F99 |
| **Substance-Related and Addictive Disorders** | 291.x (all 291 codes), 292.x (all 292 codes), 303.x (all 303 codes), 304.x (all 304 codes), 305.x. Provisional=16 | F10-19, F55, F63.0 |
| **Schizophrenia Spectrum and Other Psychotic Disorders** | 293.81, 293.82, 295.x (all 295 codes), 297.x (all 297 codes), 298.x (all 298 codes). Provisional=2 | F06.0-2, F20, F22-F29, F53.1 |
| **Mood disorders** | 293.83, 296.x (all 296 codes), 300.4x, 301.13, 311.x, 625.4. Provisional=3, 4 | F06.3, F30.x-F34.x, F38.x, F39.x, F53.0  classified |
| **Anxiety disorders** | 293.84, 300, 300.0x, 300.2x, 309.21, 313.23. Provisional=5 | F06.4, F40, F41, F93.0-2, F94.0 |
| **OCD & related disorders** | 300.3x, 300.7x, 312.39, 698.4x. Provisional=6 | F42.x, F45.2, F63.3 |
| **Personality disorders** | 301, 301.0x, 301.2x, 301.4x, 301.5x, 301.6x, 301.7x, 301.81-3, 301.89, 301.9x 310.1. Provisional=18 | F07.x (all F07 codes), F21, F60, F61, F62, F68, F69 |
| **Deliberate self-harm** | N/A (DAD/NACRS only) | X60-X84, Y10-Y19, Y28 |
| **Trauma and stressor related disorders** | 308.3x, 309, 309.0x, 309.24, 309.28, 309.3x, 309.4x, 309.81, 309.89, 309.9x, 313.89. Provisional = 7 | F43, F94.1, F94.2 |
| **ADHD** | 314.x | F90 |
| **Autism Spectrum Disorder^b^** | 299.x | F84.0, F84.1, F84.3, F84.4, F84.5, F84.8, F84.9 |

^a^OMHRS=Ontario Mental Health Reporting System, DAD=Discharge Abstract Database, NACRS=National Ambulatory Care Reporting System, OCD=obsessive compulsive disorder, ICD-9-CM=International Classification of Diseases, 9th Revision, clinical modification, ICD-10-CA=International Classification of Diseases, 10th Revision, with Canadian enhancements

^b^ Defined as one or more hospital admissions or ED visits with an ASD diagnostic code or two or more physician visits with an ASD diagnostic code
